# Merocytophagy is an integrin-stabilized macrophage response to microbes reliant on Syk signaling

**DOI:** 10.3389/fimmu.2025.1565250

**Published:** 2025-04-17

**Authors:** Kelly N. Deobald, Shaun P. Steele, Sedelia R. Dominguez, Shannon Whiles, Thomas Kawula

**Affiliations:** Allen School for Global Health, Washington State University, Pullman, WA, United States

**Keywords:** trogocytosis, macrophage, Syk, bacterial pathogens, integrin alpha 4, integrin beta 2, ICAM-1, innate immunity

## Abstract

Macrophages and dendritic cells acquire bacteria and cytosolic content from other cells without killing the donor cell through a trogocytosis-associated process termed merocytophagy. While characteristics of this behavior have been partially identified, the mechanism and potential contribution to the response to infection are unclear. Here, we reveal that a wide range of distinct species of bacteria stimulate enhanced merocytophagy in macrophages through pattern recognition receptor (PRR). Further, we found that cell-to-cell transfer in response to *Francisella tularensis* infection occurs in a predominantly MyD88-independent manner, relying on spleen tyrosine kinase (Syk) activity. Syk signaling during this response also results in increased surface expression of cell-to-cell adhesion proteins integrin α4, integrin β1, ICAM-1 and CD44 at the site of merocytophagy transfer, and depleting these surface molecules impairs merocytophagic cell-to-cell transfer. Altogether, our data demonstrate that merocytophagy is a host response to infection facilitated by tight cell-to-cell binding which molecularly resembles an immunological synapse between macrophages.

## Graphical Abstract

Image created using elements from NIAID Visual and Medical Arts, Proteins Collection. Courtesy of NIAID BIOART Source (https://bioart.niaid.nih.gov/).

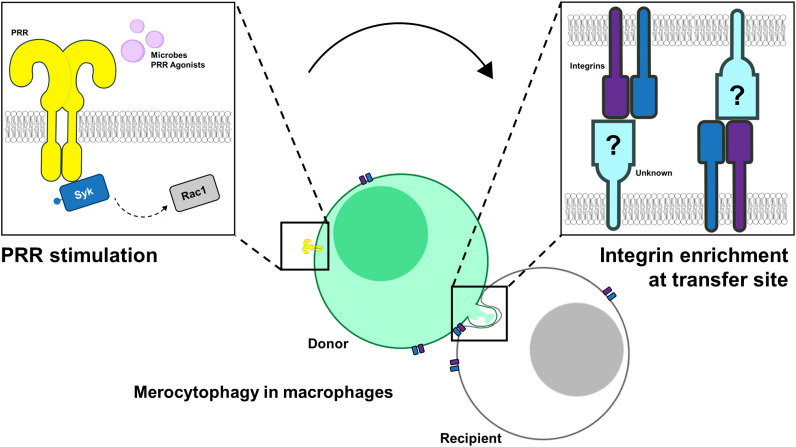

## Introduction

Cells such as dendritic cells (DCs) and macrophages mediate innate immune responses to infection and participate in the development of adaptive immune responses by processing and presenting antigens to T lymphocytes. Research on the mechanisms by which these antigen presenting cells (APCs) acquire immunogenic material has primarily focused on acquisition of antigen from the extracellular space. These acquisition mechanisms include phagocytosis, stimulation of cell-surface receptors via pathogen-associated molecular patterns (PAMPs), and active invasion by pathogens. Additional research has identified several mechanisms that involve acquisition of immunogenic material directly from infected neighboring cells (1–7). These routes include cellular exchange of antigen-containing vesicles (8), and DC antigen cross dressing, in which DCs acquire antigen-MHC complexes from other cells to decorate their own cell surface (9, 10). It has also been shown that viruses can be bundled with immune signals, so newly infected cells can respond more promptly when the genomic elements are exposed (11). These studies suggest that the host employs multiple intricate signaling mechanisms to respond to intracellular pathogens and that these processes are critical for proper immune function. However, relatively little is known about these mechanisms and their role in the response to bacteria. Here, we focus on one noncanonical mechanism, merocytophagy, a process defined by the acquisition of immunogenic material directly from the cytosol of a neighboring cell (6) and its role in the response to *Francisella tularensis* and other bacterial species.

*F. tularensis* is a highly virulent bacterium which replicates in the cytosol of phagocytes and causes the disease tularemia. Although Gram-negative, its lipopolysaccharide (LPS) does not trigger classic host toll-like receptor 4 (TLR4) signaling and subsequent inflammation due to atypical acylation patterns. This characteristic, together with its intracellular replication strategy and factors that suppress inflammatory cytokine responses in macrophages and DCs, make *F. tularensis* an immunologically silent pathogen (12).

We reported that macrophages acquire viable *F. tularensis* bacteria directly from the cytosol of neighboring cells through the trogocytosis-like process merocytophagy and demonstrated that transfer by this route is enhanced by *F. tularensis* infection (6, 13). Similar observations have been made with a wide range of intracellular pathogens, including *Plasmodium falciparum*, *Mycobacterium marinum*, and *ΔactA Listeria monocytogenes*, a mutant with defective bacteria-mediated cell-to-cell transfer (2, 4, 7). The latter two studies provide evidence that bacteria exploit host-mediated transfer as a means of expanding the replicative niche *in vivo* (2, 4, 13, 14). These reports also suggest that transfer via merocytophagy plays a role in pathogenesis, but its role remains unclear and very little is known about the mechanism of transfer.

The studies described herein investigate the process of merocytophagy and highlight this behavior as part of the immunological response to microbes. Merocytophagy is not merely an alternative mode of bacterial dissemination, as we show that cytosolic transfer by this mechanism is host-mediated and is up-regulated in response to infection and exposure to PAMPs. Here we identify a subset of PRRs which stimulate cytosolic transfer and the associated noncanonical downstream signaling pathway. Lastly, our observations demonstrate that signaling leads to increased expression of integrins which are necessary for efficient cytosolic transfer to occur, suggesting the formation of an immunological synapse as part of transfer.

## Results

### Microbial infection enhances transfer by merocytophagy

**Figure 1:**
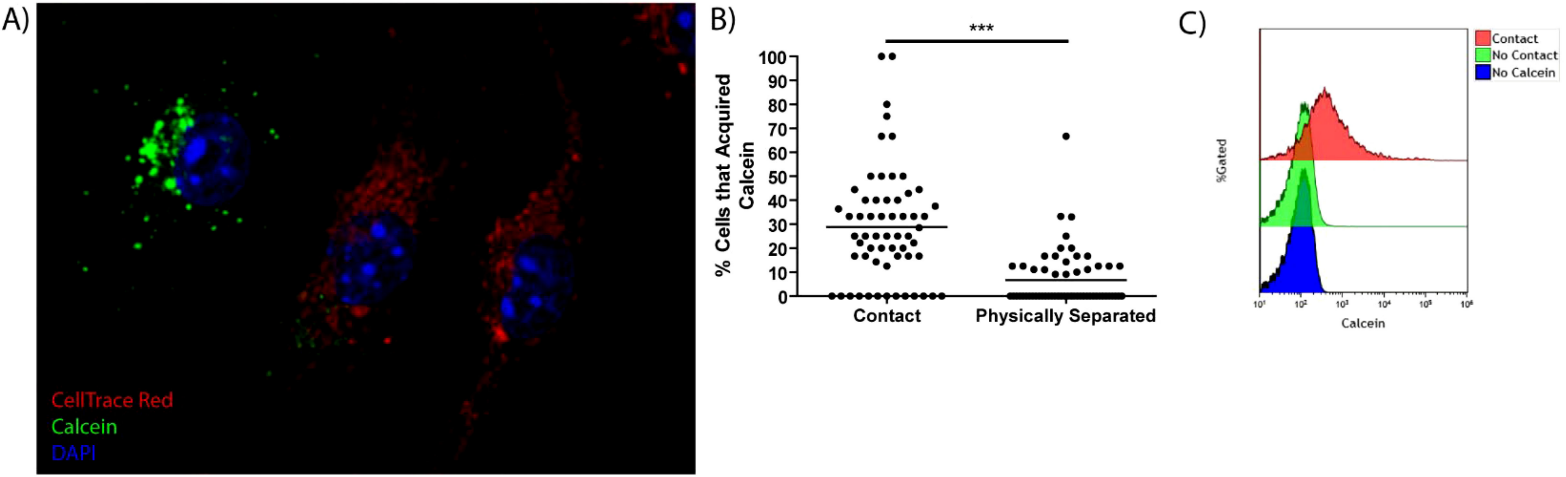
Cell-to-cell contact is required for efficient Calcein transfer in macrophages. **(A)** A Representative image of a CTR stained cell (red) that acquired Calcein (green). The nucleus is stained with DAPI (blue). **(B)** Calcein-labeled BMDMs were co-cultured with, or physically separated from CTR cells using a Transwell insert system. 20 fields of view per experiment, 3 independent experiments. Unpaired t-test. ***p<0.0001. **(C)** Representative flow cytometry histogram of Calcein transfer to CTR labeled cells when the cells are in contact or separated by Transwell system. Representative of 3 independent experiments.

We have previously shown that *F. tularensis* dissemination by merocytophagy, is a contact-dependent process (6, 13). This process differs from trogocytosis primarily in the transfer of cytosolic content and the formation of a characteristic double-membraned vacuole (6). To further investigate the mechanism of transfer, we stained bone marrow-derived macrophages (BMDMs) with the dye Calcein-AM to establish a baseline of transfer an intracellular viability dye that uniquely diffuses into the surrounding media when a stained cell loses membrane integrity (15). We added Calcein-stained BMDMs to a recipient population of CellTrace Far Red (CTR)-labeled BMDMs and measured how much Calcein transferred from donor to recipient cells (**Figure 1A**). The measured Calcein transfer from untreated cells established the baseline level of merocytophagy and any increase in Calcein transfer was interpreted as enhanced merocytophagy throughout studies. Importantly, corroborating our previous studies, BMDMs only transfer detectable levels of Calcein upon cell-to-cell contact (**Figure 1B**). This contact-dependence was also noted when assessing Calcein acquisition in CTR-labeled populations via flow cytometry (**Figure 1C**). Additionally, trypsinizing donor or recipient cells prior to co-incubation decreased the rate of merocytophagy, suggesting that trypsin-sensitive surface proteins contribute to transfer (data not shown).

**Figure 2:**
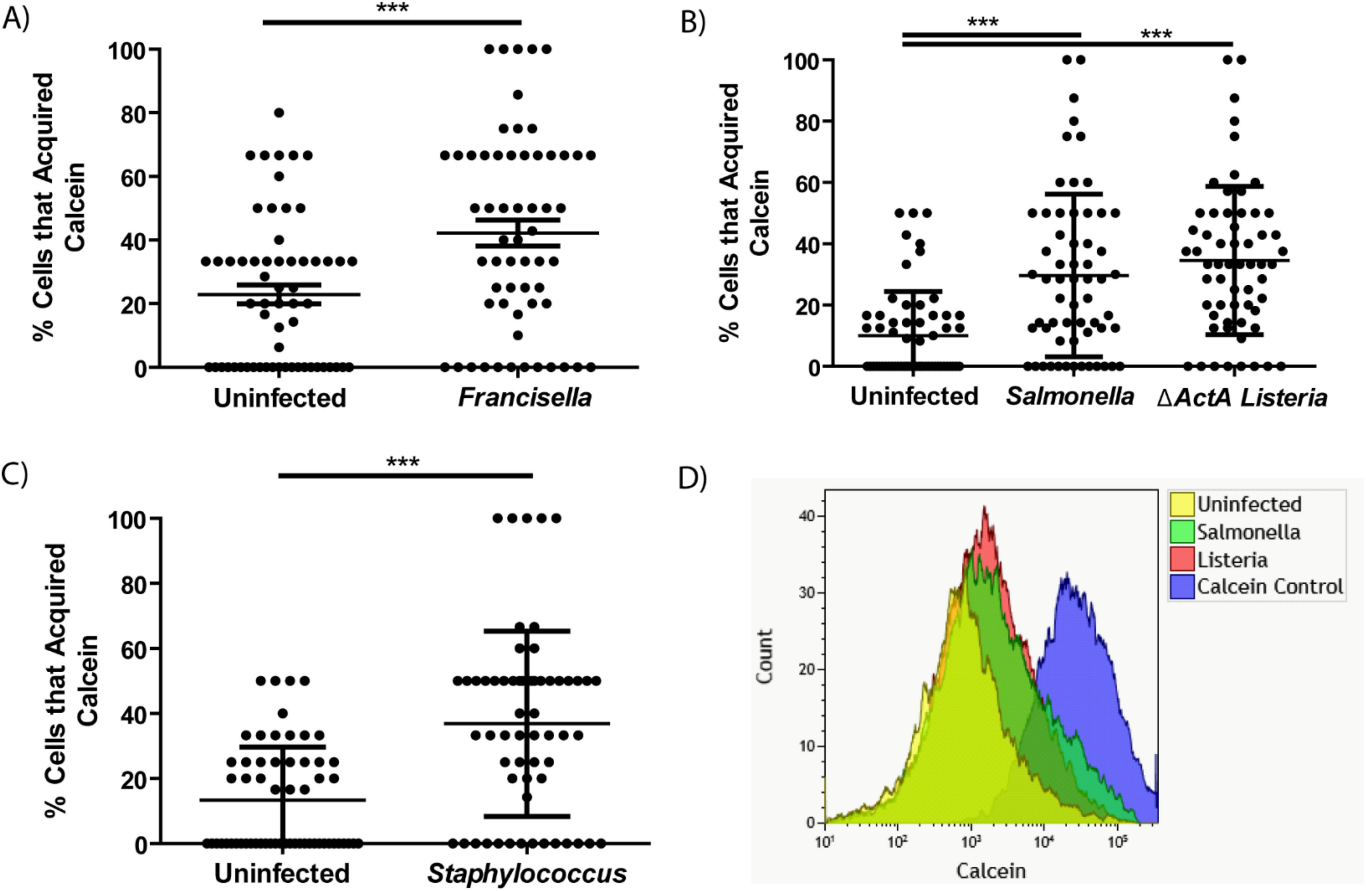
Calcein transfer between macrophages is enhanced by microbial infection. **(A-C)** The percent of CTR-labeled BMDMs in a given field of view that acquired Calcein during infection with *F*. *tularensis* **(A)**, *S. Typhimurium* **(B)**, *L. monocytogenes* **(B)**, or *S. epidermidis* **(C)**, as indicated. Mean +/- SD, minimum of 3 CTR-stained recipient cells per field of view quantified and 20 fields of view imaged for each experiment. Results of 3 independent experiments. Panel A and C analyzed by unpaired t-test, panel B analyzed by one-way ANOVA with Dunnett post-test. ***p <0.0001. **(D)** Overlay of representative flow cytometry data assessing Calcein positivity in CTR-labeled recipient BMDMs where the donor population was Calcein stained and infected with the indicated pathogen. Performed in triplicate per pathogen.

Merocytophagy has been shown to occur during infection with a wide range of pathogens with diverse replication strategies (2, 4, 13). To assess the relationship between merocytophagy and microbes, we infected donor populations of BMDMs with a variety of bacterial species and stained with Calcein prior to contact with CTR-labeled recipient cells. Bacterial species for donor infection were chosen to represent broad classes of organisms with diverse replication strategies. *F. tularensis* represented cytosolic Gram-negative and *Listeria monocytogenes* accounted for cytosolic Gram-positive pathogens. The specific *ΔactA Listeria* mutant used for experiments is deficient in actin polymerization activity that is required for *Listeria*-mediated cell-to-cell transfer, limiting this organism to merocytophagy-mediated dissemination. Lastly, *Salmonella enterica* serovar Typhimurium is vacuolar in macrophages and *Staphylococcus epidermidis* is an extracellular, commensal bacterium. We found that recipient BMDMs acquired significantly more Calcein in the context of bacterial infection compared to uninfected BMDMs (**Figures 2A–C**). Enhanced Calcein transfer during infection was also contact-dependent (**Supplementary Figure 1**) and infection with each bacterial species significantly increased both the number of cells that acquired Calcein and the mean Calcein dye acquired per CTR-labeled recipient cell (**Figure 2D**; **Supplementary Figure 2**). These results establish that the infection-enhanced merocytophagy shown previously (6, 13) is not unique to *F. tularensis* infection, providing support for merocytophagy as a host-mediated response.

Not only do a variety of bacteria stimulate merocytophagy, we found that bacterial viability is not required to mediate enhanced cytosolic transfer. The supernatant of boiled *S.* Typhimurium was sufficient to stimulate increased Calcein transfer (**Supplementary Figure 3**). Altogether, the diverse range of bacteria that increase cytosolic transfer suggests that macrophages undergo merocytophagy at a baseline rate and up-regulate merocytophagy in response to microbial infection.

### Cytosolic transfer is induced by TLR4 and C-type lectin receptor signaling via the Syk pathway

**Figure 3:**
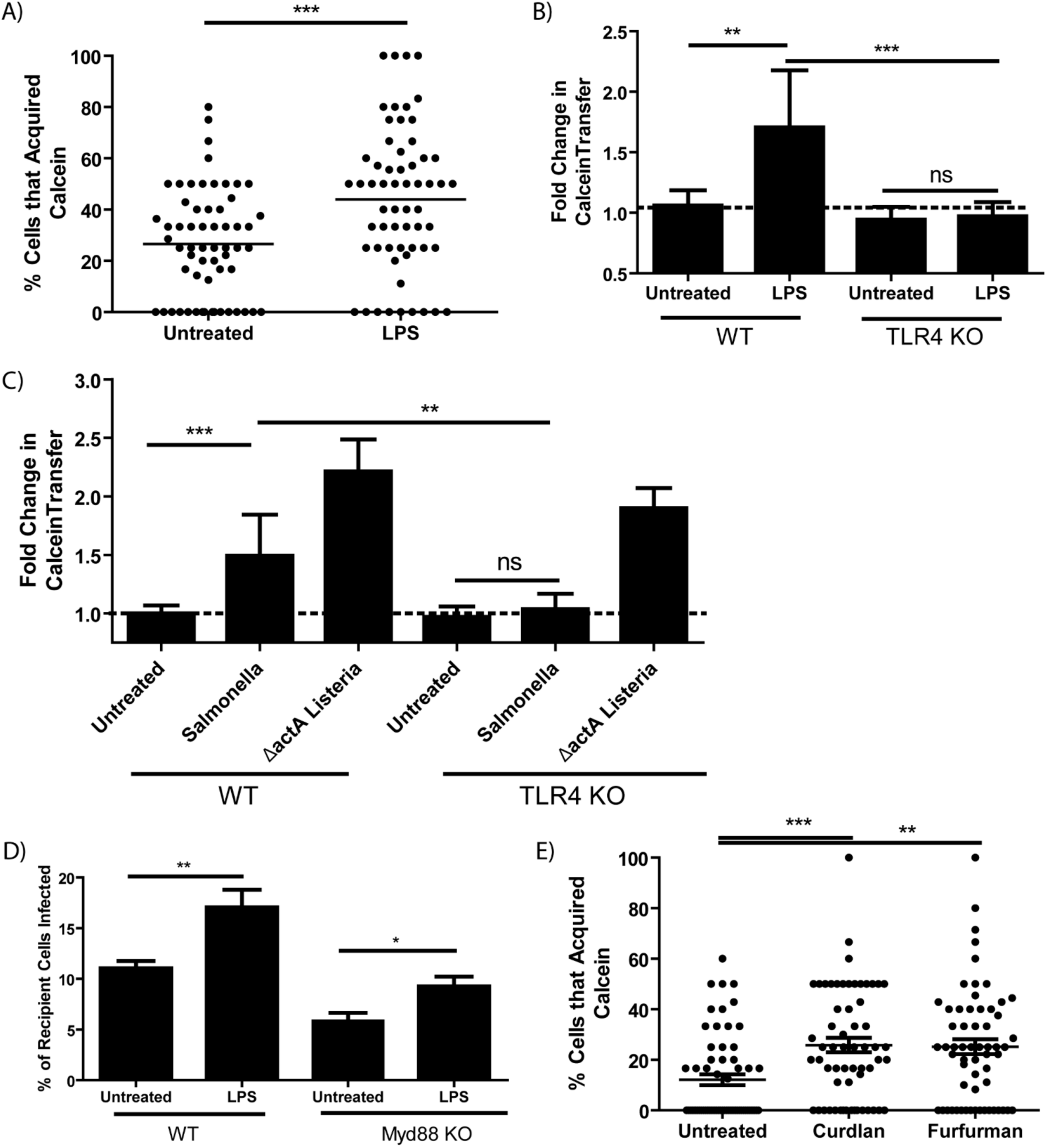
Merocytophagy is a host response to pathogens via TLR4 and CLR stimulation. **(A)** The percent of CTR-labeled BMDMs in a given field of view that acquired Calcein with or without *E*. *coli* LPS treatment (50 ng/mL). Minimum of 3 CTR-stained recipient cells per field of view with 20 fields quantified per experiment. Results of 3 independent experiments, mean+/- SD, analyzed by unpaired t-test. ***p <0.0001 **(B, C)** Calcein-labeled wildtype or TLR4 knockout BMDMs treated with *E*. *coli* LPS (50 ng/mL) **(B)** or infected with the indicated strain **(C)** were co-incubated with CTR-stained recipient BMDMs and assessed by flow cytometry. Mean +/- SD. Y-axis represents the geometric mean fluorescence intensity of Calcein in recipient cells normalized to untreated control (dotted line). Data from 3 independent experiments performed in triplicate and analyzed by unpaired t-test. Ns, not significant. **p<0.001, ***p<0.0001. **(D)** The percent of recipient cells infected by merocytophagy of *F*. *tularensis* in wildtype donor-recipient pairings and Myd88-deficient donor-recipient pairings. Assessed by bacterial staining using fluorescent anti-*Francisella* LPS antibody and flow cytometry. Mean +/- SD. Data from 3 independent experiments performed in triplicate and analyzed by unpaired t-test. **(E)** The percent of CTR-labeled BMDMs in a given field of view that acquired Calcein during cell-to-cell contact with or without treatment with the indicated Dectin agonist (Curdlan, 100 μg/mL. Furfurman, 10 μg/mL). Minimum of 3 CTR-stained recipient cells per field of view with 20 fields quantified per experiment. Results of 3 independent experiments, mean+/- SD, analyzed by one-way ANOVA with a Dunnett post-test. *p<0.05, **p<0.001, ***p <0.0001.

Since a variety of bacteria enhance cytosolic transfer, it is likely that pattern recognition receptors (PRRs) may stimulate the process. Lipopolysaccharide (LPS) is a strong agonist present in the boiled *S.* Typhimurium supernatant which stimulated cytosolic transfer (**Supplementary Figure 3**). We tested if LPS alone was sufficient to enhance Calcein transfer to CTR-labeled recipient macrophages. We found that as little as 10 ng/mL of *E. coli* LPS increased cytosolic transfer to a similar level of that observed during infection (**Figure 3A**; **Supplementary Figure 4A**) and LPS stimulation rapidly up-regulated Calcein transfer within 2 hours of treatment (**Supplementary Figure 4B**). Further, LPS-stimulated merocytophagy required TLR4 expression, with TLR4 activity also responsible for *S.* Typhimurim*-*induced merocytophagy but not transfer induced by Gram-positive *ΔactA L. monocytogenes* (**Figures 3B, C**).

We next tested merocytophagy transfer in the absence of the TLR adaptor MyD88. MyD88-deficient BMDM were infected with *F.tularensis* and Calcein-stained prior to contact with CTR-labeled recipients. Surprisingly, although MyD88-deficient pairings had decreased transfer of bacteria compared to wildtype pairings, merocytophagic dissemination was partially rescued by the addition of LPS, suggesting noncanonical TLR4 signaling for LPS-enhanced transfer (**Figure 3D**). It should also be noted that a slight infection defect was observed in MyD88-deficient BMDMs, which likely contributed to the decreased *F. tularensis* transfer (data not shown).

Of the bacterial species tested, only *S.* Typhimurium canonically stimulates TLR4, indicating that an alternative PRR pathway is involved in stimulating enhanced merocytophagy. TLR4 can signal through the same pathway as C-type lectin receptors (CLRs) which canonically signal through spleen tyrosine kinase (Syk). We therefore tested whether agonists of CLRs Dectin-1 and Dectin-2 enhance merocytophagy transfer (16, 17). Curdlan is a specific β-glucan which stimulates Dectin-1 and furfurman stimulates Dectin-2. Similar to LPS stimulation, both CLR agonists tested enhanced merocytophagy (**Figure 3E**).

**Figure 4:**
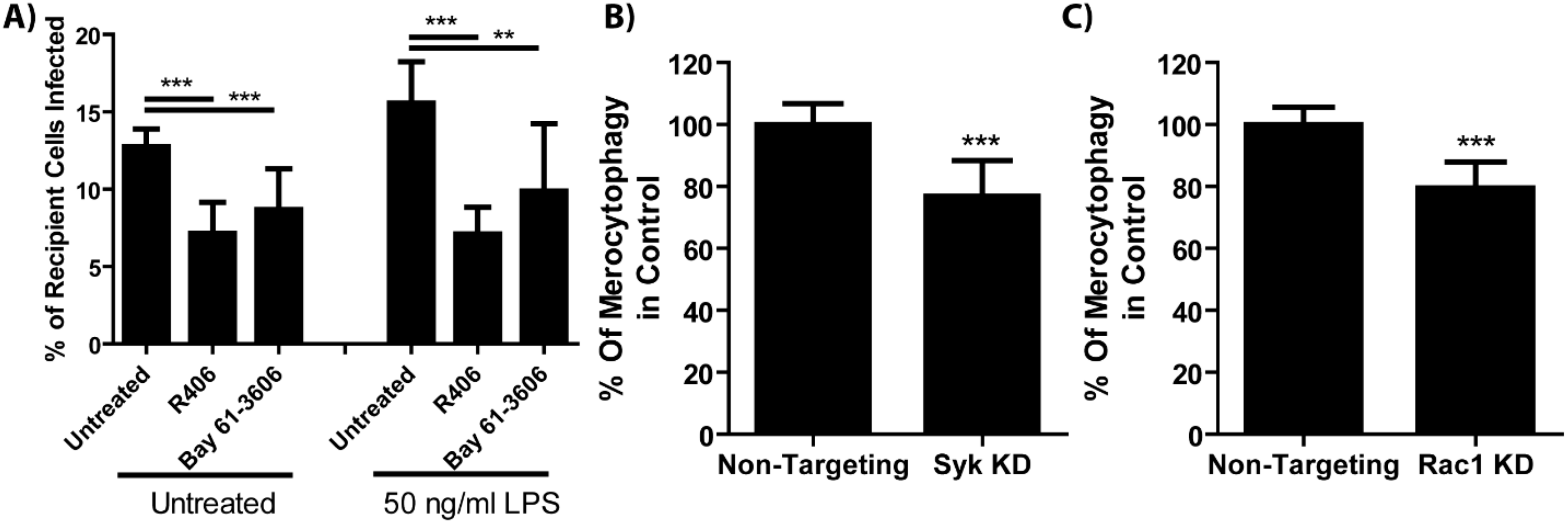
Cell-cell transfer of bacteria requires Syk and Rac1. **(A)** The fraction of recipient cell populations that became infected over 6 hours co-incubation time when the cells were treated with Syk inhibitors (R406, 10μM, or Bay 61-3606, 10 μM) with or without *E*. *coli* LPS. One-way ANOVA with a Dunnett post-test. **(B, C)** The number of recipient cells that became infected with *F*. *tularensis* normalized to the percent of donor cells that were infected. See **Supplementary Figure 6** for infection rates. Unpaired t-test. **p<0.001, ***p<0.0001 All results from 3 experiments performed in triplicate. Mean +/- SD.

Consistent with these receptor agonist results, treating *F. tularensis-*infected BMDMs with Syk inhibitors reduced *F. tularensis* transfer (**Figure 4A**). Further supporting Syk pathway activity in cytosolic transfer, we observed that in populations of J774A.1 macrophages treated with the phosphoinositide 3-kinase (PI3K) inhibitor Wortmannin had significantly fewer cells which acquired content from donors compared to vehicle-treated controls (**Supplementary Figure 5**). Likewise, BMDMs that were treated with siRNAs to knockdown Sykβ or the downstream kinase Rac1 showed decreased *F. tularensis* transfer by merocytophagy (**Figures 4B, C**). In these experiments, Rac1-knocked down donor populations had significantly more initially infected cells compared to non-targeting controls, while Sykβ-knocked down donor populations had comparable infected donor cells compared to respective non-target controls (**Supplementary Figure 6**). These results exclude poor phagocytosis or bacterial uptake in the donor cell population as the cause for decreased merocytophagy transfer. Rather, these data indicate that microbes stimulate merocytophagy via Syk and Rac1 to enhance transfer.

### Cell adhesion proteins are necessary for robust merocytophagy

**Figure 5:**
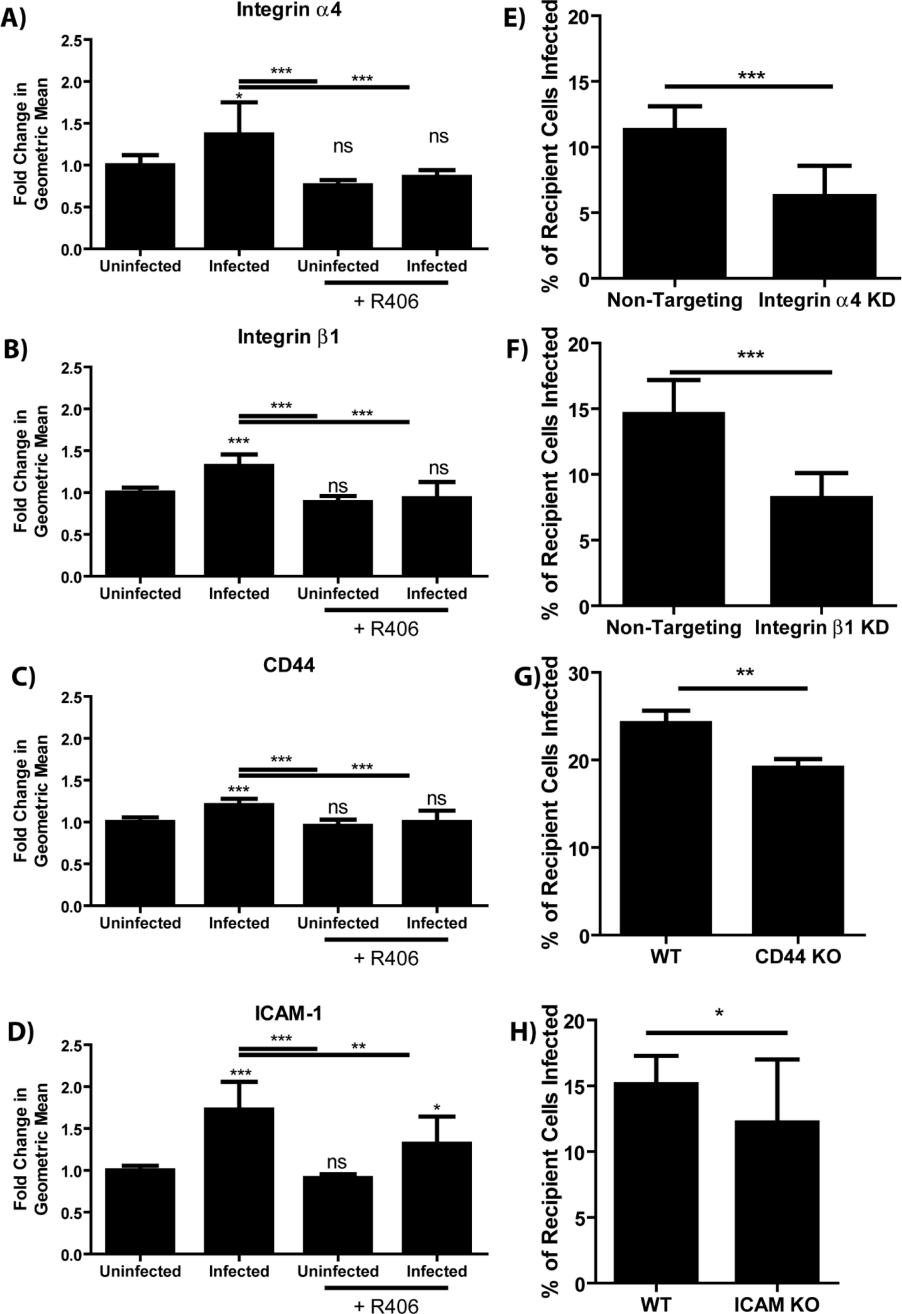
Syk enhances merocytophagy by increasing surface expression of cell adhesion proteins. **(A–D)** Relative abundance of the indicated cell adhesion molecule during *F. tularensis* infection with and without the treatment with Syk inhibitor (R406, 10μM). Analyzed using a one-way ANOVA with a Dunnett post test. Significance notations immediately above the bar are compared to the uninfected, untreated sample **(E–H)** The percent of recipient cells infected by co-incubation with infected donor population. Labels indicate both the donor and recipient population were wildtype, or that knockdown or knockout cells were used as both the donor and recipient for the appropriate experiments. Assays performed 3 times in triplicate. Mean +/- SD. Analyzed using an unpaired t-test. Panel H used a paired t-test due to batch-to-batch variation in the sensitivity of the conjugated *F. tularensis* antibody. See Materials and Methods. Ns, not significant, *p<0.05, **p<0.001, ***p<0.0001.

Given that merocytophagy is enhanced by bacterial infection, we assessed surface protein alteration during infection as a strategy to identify those involved in merocytophagy. Theoretically, infected cells which upregulate merocytophagy do so by upregulating proteins which aid in facilitating transfer or by decreasing expression of merocytophagy-suppressing proteins. To test this, we trypsinized the surface of *F. tularensis-*infected or uninfected J774A.1 macrophages and analyzed protein fragments by mass spectrometry (**Supplementary Table 1**). Within this screen, we identified 4 cell adhesion proteins that were both increased in the infected samples and known to interact with the Syk pathway. These proteins were integrins α4 and β1, which form the heterodimer VLA-4, as well as ICAM-1 and CD44 (18–20). The up-regulation of these proteins on the cell surface of infected BMDMs was further validated by flow cytometry (**Figures 5A–D**). Additionally, treating the infected BMDMs with the Syk inhibitor R406 during the last 6 hours of infection significantly reduced surface expression of these integrins, indicating that cells up-regulate these proteins in response to *F. tularensis* infection via Syk activation (**Figures 5A–D**).

To confirm that these cell surface proteins contribute to merocytophagy in primary cells, we used siRNAs to knock down integrin α4 or integrin β1 in populations of BMDMs and verified decreased expression via flow cytometry (**Supplementary Figure 7**). Cells with decreased surface expression of these integrins exhibited reduced transfer of *F. tularensis* by merocytophagy compared to cells treated with non-targeting control siRNAs (**Figures 5E, F**). Similarly, BMDMs from mice with genetic knockouts of ICAM-1 or CD44 also showed impaired *F. tularensis* transfer (**Figures 5G, H**). Interestingly, ICAM-1 was previously identified for playing a similar role in plasmacytoid DCs (1). Taken together, these cell-to-cell adhesion molecules are all required for optimal merocytophagy.

Our data indicate that integrin α4, integrin β1, ICAM-1, and CD44 individually contribute to merocytophagy. However, the absence of any one of these cell adhesion molecules is likely compensated by others with overlapping functions. For all integrin depletion assays, we found that both the donor and recipient must lack the target protein before decreased merocytophagy is observed.

### Cell-to-cell adhesion proteins localize to the site of cytosolic transfer

Interestingly, three of the four proteins identified in the screen are known to function in the tight cell-to-cell binding within the immediate periphery of T cell immunological synapses, as part of the peripheral supramolecular activation cluster (pSMAC) (18, 21, 22). Additionally, Zap70 is a specific Syk kinase required for coordination of this canonical immune synapse, further highlighting a potential role for merocytophagy within the broader immune response (23). We therefore hypothesized that phagocytes participating in merocytophagy utilize the same proteins for tight cell-to-cell interactions in the same fashion to the immune synapse.

**Figure 6:**
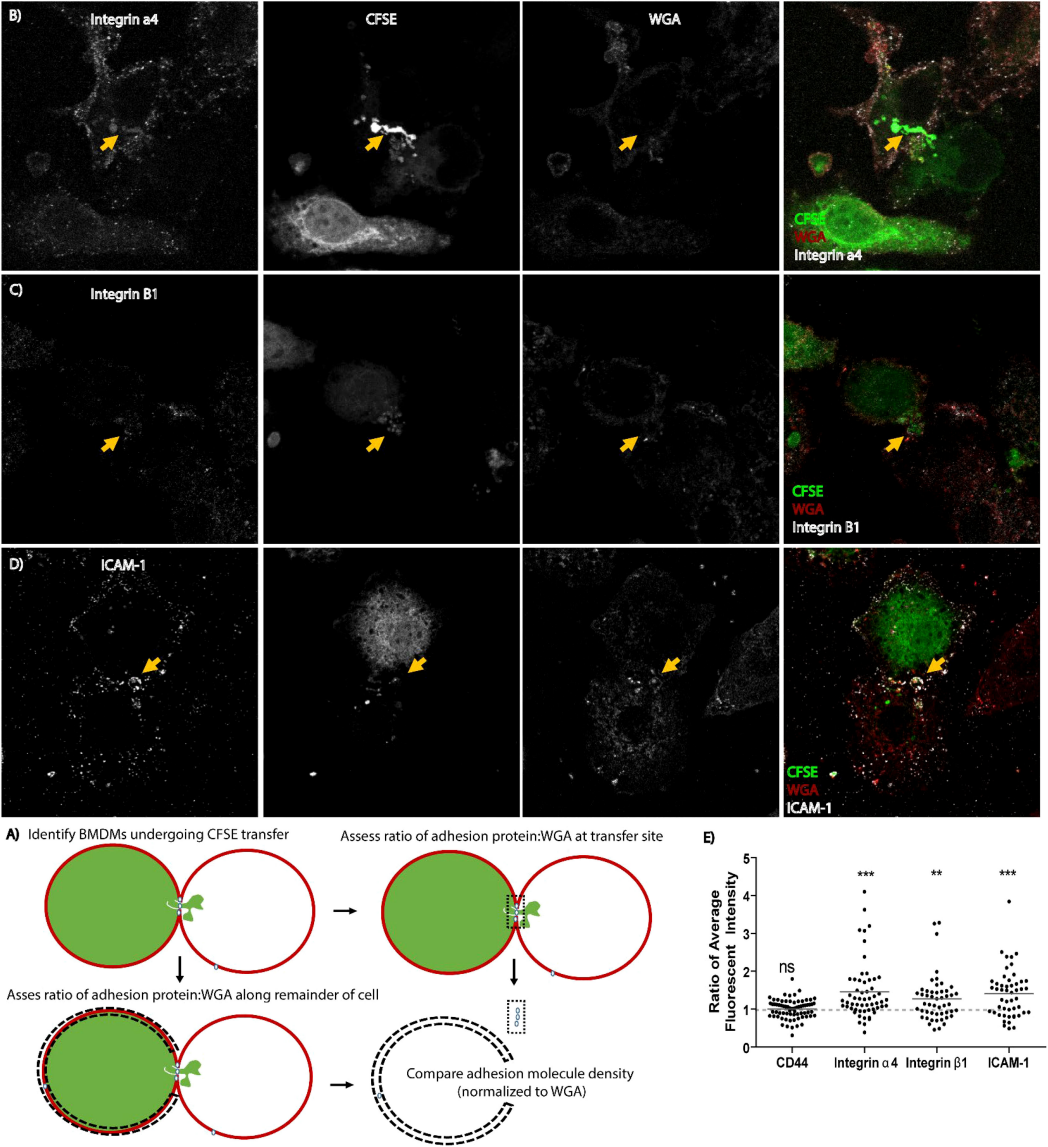
Cell adhesion molecules re-localize to the site of merocytophagy. **(A)** Diagram of how microscopy analysis was performed. **(B–D)** Representative maximum projection microscopy images of BMDMs exchanging CFSE in unstimulated cells. The yellow arrow indicates increased protein aggregation at the site of CFSE transfer. **(E)** Results of re-localization of the indicated cell adhesion molecule to the synapse during transfer of CFSE. Each dot represents a cell. All cells identified from 3 independent experiments included in analysis. Total cell count for each adhesion molecule assessed: integrin α4- 57 cells, integrin β1- 50 cells, CD44- 70 cells, ICAM-1- 50 cells. One sample t-test with a hypothetical value of 1. Ns, not significant, **p<0.001, ***p<0.0001.

To test this hypothesis, we stained a donor population of BMDMs with carboxyfluorescein succinimidyl ester (CFSE), which nonspecifically dyes proteins throughout the cell, then added an unstained recipient population of BMDMs for 1 hour. Integrins were stained using primary monoclonal antibodies and fluorescently tagged secondary antibodies and cell-to-cell interactions were examined by confocal microscopy to test for enrichment of ICAM-1, CD44, integrin α4 or integrin β1 at the site of CFSE transfer. For this assessment, we normalized the mean fluorescence intensity of the antibody signal against intensity of signal from lectin wheat germ agglutinin (WGA) tagged with a fluorochrome. WGA stains plasma membrane of cells and this internal staining control accounts for donor and recipient cell membrane present at the cell-to-cell interface and any other variations in membrane content. Staining intensity of integrins relative to WGA staining at the transfer site was assessed in comparison to staining intensity in equivalent sections of membrane along the same cell (**Figure 6A**).

We observed that ICAM-1, integrin α4, and integrin β1, which are known pSMAC components; were enriched at the site of CFSE transfer in both donor and recipient cells (**Figures 6B–D**). In contrast, CD44 did not appear to have altered localization on the cell during merocytophagy (**Figure 6E**). These results are consistent with the formation of a pSMAC-like complex at the site of merocytophagy with CD44 likely contributing to cell-to-cell binding along the entire cell-to-cell interface.

## Discussion

While bacteria can exploit merocytophagy as a cytosolic transfer process for dissemination, this process appears to be host-mediated. Macrophages acquire fluorescent beads and cytosolic dyes from neighboring cells in the absence of infection (13). We have previously demonstrated that merocytophagy alone does not neutralize *F. tularensis* infection, as bacteria escape the merocytophagic vacuole to replicate in the cytosol of recipient macrophages (6). However, it is unclear how commonly microbes survive following merocytophagy-mediated transfer between cells, especially APC recipients. There is some evidence that for a subset of pathogens, the transfer process could be detrimental to the microbe (1). Related work in the context of viral infections suggest that transfer of viruses or viral antigens induce an interferon response from recipient plasmacytoid DCs, which destroys the virus (1). The authors found that this process was also contact and ICAM-1-dependent, suggesting that the mechanism for this process may be similar to bacterial transfer by merocytophagy. Notably, shared signaling pathways involving Rac1, Syk, and Arp2/3 also suggest potential mechanistic similarities between bacterial transfer by merocytophagy and viral transfer responses in plasmacytoid DCs (1, 24, 25). Although preliminary data showed that stimulating TLR7/8 using purified agonist R848 was not sufficient to enhance transfer in our model, there is the possibility that additional cytosolic factors bundled with virus could induce merocytophagy (11) and this observation warrants further investigation into the broader role of merocytophagy in host-pathogen interactions. Additionally, more work is necessary to investigate the contribution of different TLRs to the enhanced phenotype reported, as our findings focus on TLR4 as well as other PRRs which signal primarily via Syk. More in-depth studies on TLR4 stimulation and signaling, as well as studies investigating cells singly- or doubly- deficient for specific TLRs and their signaling mediators, offer a possible route to provide a greater understanding of merocytophagy transfer and the mechanism behind it.

Providing further evidence of shared signaling mechanisms, a significant contribution of actin-polymerization machinery was recently demonstrated in *F. novicida* merocytophagy transfer between J774A.1 cells, as pharmacologic inhibition via Cytochalasin D and Latrunculin A together decreased bacterial transfer by 84.6% (26). In conjunction with our findings, which demonstrate *F. tularensis* transfer in BMDMs is facilitated in part through Rac1 signaling downstream of Syk activity (**Figure 4C**), this suggests that actin-polymerization is common to *Francisella* bacterial transfer among multiple subspecies. Interestingly, the authors also observed *F. novicida* concentrated near the site of transfer, suggesting that cell polarization contributes to merocytophagy (26).

While the receptor or signaling pathway directly responsible for triggering merocytophagy remains unknown, our data demonstrate a connection to the Syk signaling pathway. Interestingly, it was recently established that the TLR adaptor MyD88 interacts directly with Syk to activate NF-κB in LPS-stimulated RAW263.7 macrophages (27). The Syk signaling pathway has also been implemented in MHC-II cross-dressing in a basophil model (28), expanding on the importance of Syk activity in trogocytosis-like behaviors and outlining a need for additional research on the effect of signaling on innate cell functions. Overall, the involvement of Syk highlights the potential for merocytophagy to contribute to the immune response against a variety of bacteria, as demonstrated here, as well as viruses and other immunogenic reactions.

Interestingly, neutrophils ingest cytosolic material from opsonized tumor cells, resulting in eventual death of the tumor cell (29). This process bears resemblance to the transfer of bacteria in macrophages demonstrated here. However, while neutrophils swarm tumor cells to mediate many cytosolic transfer events to facilitate tumor cell killing (29), we have observed that cytosolic acquisition events during macrophage merocytophagy does not kill the donor (13). Instead, cytosolic acquisition of bacteria by macrophages appears to prolong the life of the infected cell (**Supplementary Figure 8**). It is also notable that, based on the similarities identified by microscopy at the molecular level, bacterial acquisition by macrophages and cytosolic transfer-induced killing by neutrophils likely occurs through similar mechanisms. Conversely, the two processes are potentially regulated in contrary ways. This difference points to the possibility that merocytophagy transfer is differentially regulated in different cell types to lend nuance to cell functions. Like the study conducted by Matlung et al., trogocytosis of tumor cells has been observed in other granulocytes, namely eosinophils and basophils (30). In support of our observations reported here, the authors proposed a model which included an immunological synapse, although CD18/CD11b were reported as the contributing stabilizing integrins (29, 31). In contrast to our studies, neutrophil mediated trogocytosis of tumor cells depends heavily on antibody opsonization of the target and can be inhibited by expression of sialic acids on the target surface (31, 32). Altogether, these observations highlight similar trogocytosis-like behaviors which may be carried out by cell type-specific factors and machinery. Further, the cell adhesion factors we and others found appear to be critical for merocytophagy to occur efficiently (1, 28). Most likely, the role of these factors is to prolong cell-to-cell contact time to facilitate merocytophagy more frequently or successfully. However, we cannot rule out the possibility that these factors serve to regulate the quantity of material phagocytosed, or even trigger signaling cues to initiate transfer in specific cell types (33). Elucidating the precise functions of signaling and cell surface factors in merocytophagy in macrophages and other cell types represents a promising avenue for future research.

Concisely, our work establishes that merocytophagy is a host response to pathogens. This process is enhanced by PRR signaling and largely dependent on the Syk signaling pathway, ultimately increasing surface expression of cell adhesion molecules, namely integrin α4, integrin β1, ICAM-1 and CD44 to form a synapse-like structure and facilitate transfer of bacteria and cytosolic content.

## Materials and methods

### BMDM cultures, knockouts and knockdowns

Bone marrow-derived macrophages (BMDMs) were generated by incubating bone marrow cells from 6-10 week old C57Bl/6J (wildtype, **Table 1**) mice for 6 days in DMEM supplemented with 20% fetal bovine serum (FBS), 30% L929 conditioned media, sodium bicarbonate, 1mM sodium pyruvate, and 1% GlutaMax (ThermoFisher, 35050061). Macrophage identity was confirmed by flow cytometry to assess surface expression of CD11b (Biolegend, clone M1/70), CD11c (Biolegend, clone N418), F4/80 (Biolegend, clone BM8), MHC-I (Biolegend, clone SF1-1.1) and MHC-II (eBioscience, clone M5/114.15.2). Knockout BMDMs (ICAM-1, CD44) were generated as above from genetic knockout mice (Jackson Labs) in a C57BL/6J background. For siRNA knockdowns (Sykβ, RAC1, integrin α4, integrin β1) wildtype BMDMs were transfected with siRNA using RNAiMax lipofectamine and the indicated targeting siRNA in Optimem according to the manufacturer’s protocol for 72 hours prior to experiments. Transfection media was replaced with fresh media just prior to experiments. Sykβ and Rac1 knockdown was confirmed by RT-PCR prior to experimentation and integrin α4, integrin β1 was confirmed by flow cytometry (**Supplementary Figure 7**).

**Table 1:** Reagents and catalog numbers used in research.

Critical Reagents			
Resource	Source	Catalog Number	Notes
*Francisella tularensis tularensis* strain *Schu S4*	BEI Resources		
*Francisella tularensis holartica Live Vaccine Strain*	CDC		
*Francisella tularensis holartica Live Vaccine Strain ΔdotU inducible dotU (required for T6SS formation)*			(Steele et al., 2019) (6)
*Francisella tularensis holartica Live Vaccine Strain inducible dotU (wildtype control)*			(Steele et al., 2019) (6)
*Salmonella enterica* serovar Typhimurium	a gift from Ed Miao		
*ΔactA Listeria monocytogenese*	a gift from Ed Miao		
*Staphylococcus epidermidis*			(Hobbs et al., 1998) (34)
anhydrous tetracycline	Cayman Chemicals	100009542	
C57BL/6J mice	Jackson Labs	000664	
CD44 knockout mice	Jackson Labs	005085	
ICAM-1 knockout mice	Jackson Labs	002127	
Myd88 knockout mice	Jackson Labs	009088	
TLR4 knockout mice	Jackson Labs	029015	
J77A.1 macrophages	ATCC	TIB-67	
Pooled non-targeting control siRNA	Dharmacon	D-001810-10-05	
Pooled Integrin α4 siRNA	Dharmacon	L-043758-02-0005	
Pooled Integrin β1 siRNA	Dharmacon	L-040783-01-0005	
Pooled SykB siRNA	Dharmacon	L-041084-00-0005	
Optimem	Gibco	31985088	
Recombinant mouse M-CSF	Peprotech	315-02	
Lipofectamine RNAiMax	ThermoFisher	13778075	
Calcein-AM	Corning	354216	1:1000 dilution
CellTrace Far Red	Invitrogen	C34564	1:1000 dilution
CFSE	Invitrogen	C34554	1:1000 dilution
*Escherichia coli* O111:B4 LPS	Sigma	L2630	used at 50 ng/mL
Curdlan	Invivogen	tlrl-curd	used at 100 µg/mL, does not go into solution
Furfurman	Invivogen	tlrl-ffm	used at 10 µg/mL
R406	Cayman Chemicals	11422	used at 10 μM
Bay 61-3606	Cayman Chemicals	11423	used at 10 μM
AF 647 anti-CD49d (integrin α4) antibody	Biolegend	103613	1:200 dilution
AF 488 anti-CD29 (integrin β1) antibody	Biolegend	102212	1:200 dilution
AF 647 CD44 antibody	Biolegend	103017	1:200 dilution for flow, 1:75 dilution for microscopy
AF 647 CD54 (ICAM-1) antibody	Biolegend	116111	1:200 dilution
anti-CD49d (integrin α4) antibody	Biolegend	103707	1:100 dilution
anti-CD29 (integrin β1) antibody	Novus Biologicals	NBP2-36561	1:50 dilution
CD54 (ICAM-1) antibody	Biolegend	116109	1:50 dilution
TRITC wheat germ agglutinin	Invitrogen	W849	1:200 dilution
AF647 wheat germ agglutinin	Invitrogen	W32466	1:200 dilution
TRITC anti-Rat secondary antibody	eBiosciences	26-4826-82	1:500 dilution
TRITC anti-mouse secondary antibody	Invitrogen	A16071	1:500 dilution
AF 647 anti-Rat secondary antibody	Invitrogen	A21247	1:500 dilution
AF 647 anti-mouse secondary antibody	Invitrogen	A21235	1:500 dilution
anti-CD11b antibody	Biolegend	101223	1:200 dilution
anti-CD11c antibody	Biolegend	117314	1:200 dilution
anti-F4/80 antibody	Biolegend	123109	1:200 dilution
anti-MHC-I antibody	Biolegend	116615	1:200 dilution
anti-MHC-II antibody	eBioscience	12-5321-82	1:200 dilution
Pierce Cell Surface Protein Isolation Kit	ThermoFisher	89881	
anti-LAMP-1 antibody	Developmental Studies Hybridoma Bank	Clone 1D4B	1:1000 dilution
anti *Francisella* LPS antibody	US Biologicals	F6070-02X	Conjugated with fluorophores in house

### Bacterial cultures

*Francisella tularensis* strains were cultured on chocolate agar supplemented with 1% isovitalex. After 3 days, 4-6 isolated colonies were combined and grown overnight in Chamberlain’s defined media (35). *S.* Typhimurium*, S. epidermidis*, and *L. monocytogenes* were cultured on Luria-Bertani (LB) agar and then single colonies were grown overnight in LB broth.

### Bacterial infections

BMDMs were infected with *F. tularensis, S.* Typhimurium*, L. monocytogenes*, at multiplicities of infection (MOIs) indicated below for 2 hours then treated with gentamicin as follows: *F. tularensis* infection MOI 100 with 10 μg/mL gentamicin added post infection and present through duration of experiment (24 hours total). Virulent SchuS4 strain was used in all *F. tularensis* experiments. *S.* Typhimurium (MOI 10) and *ΔactA L. monocytogenes* (MOI 0.05) were treated with 50 μg/mL gentamicin for one hour post infection, then incubated with 10 μg/mL through experiment (10 hours total). Cells exposed to *S. epidermidis* (MOI 4) for duration of experiment (8 hours total) with no antibiotics.

### Calcein transfer co-incubation assays

For flow cytometry, 100,000 BMDMs were infected in 24-well plates. Donor populations were stained with Calcein-AM and/or infected with indicated bacterial species as described above and recipient populations were stained with CellTrace Far Red (CTR) 1 hour before co-incubation. After staining, cells were washed and co-incubated for 6 hours. Cells were then scraped from wells and fixed in 4% paraformaldehyde (PFA) for immediate flow cytometry analysis. For microscopy, 200,000 BMDM donors were infected with indicated bacterial species on coverslips before co-incubation with CTR-stained recipients for 6hours on the coverslips. Following co-incubation, media was removed and replaced with 4% PFA for 15 minutes. Coverslips were mounted in DAPI containing mounting media for imaging by confocal microscopy. In the case of *F. tularensis* infection studies, 125,000 or 250,000 BMDM recipients were added to account for overnight cellular replication in the infected population to generate a 1:1 donor-recipient ratio in flow cytometry and microscopy experiments, respectively.

For the boiled *S.* Typhimurium experiment, 1 mL of a *S.* Typhimurium overnight culture was pelleted in 500 μl of PBS and boiled for 30 minutes. The sample was centrifuged at approximately 21,000 xg for 5 minutes. 50 μl of the soluble supernatant was added at the start of donor-recipient co-incubation.

For the TLR4 and CLR agonist experiments, 50 ng/mL of *E. coli* lipopolysaccharide (LPS), 10 μg/mL furfurman or 100 μg/mL curdlan were added at the start of co-incubation.

For physical contact control assays, recipients were stained with CTR on coverslips in 12 well plates or on plastic of 24 well plates for flow cytometry as above. Calcein-labeled or *F. tularensis*-infected donor cells were stained and washed in the top compartment of Transwell inserts in separate wells. After staining, donor and recipient populations were washed and Transwell inserts were placed over the CTR-stained recipients and co-incubated for 6 hours.

### *Francisella* transfer assays

BMDMs were seeded and infected with *F. tularensis* for 2 hours as in Calcein transfer co-incubation assays described above. 18 hours post-infection, CTR-labeled recipient cells, as well as Syk inhibitors or *E. coli* LPS where indicated, were added with fresh media and gentamicin. At 24 hours post-infection (6 hours of co-incubation), BMDMs were scraped, stained with surface antibody where indicated, and fixed in 4% PFA. Finally, cells were permeabilized in with 0.1% saponin and 2% FBS in PBS and stained with anti-*Francisella* LPS antibody conjugated in house. Cells were analyzed by flow cytometry for *F. tularensis* presence in CTR-labeled recipient cells. ***Note:*** we experienced minor batch-to-batch variation in the sensitivity of the antibody conjugation. With the exception of Panel H in **Figure 5**, all experiments used the same batch of antibody to reduce the impact of conjugation efficiency on our interpretations.

### Re-localization of integrins

BMDMs labeled with CFSE or CTR were co-incubated for 1 hour on coverslips. Cells were fixed in 4% PFA, then washed and stained with fluorescently-tagged wheat germ agglutinin (WGA) followed by extracellular staining of the indicated antibody. All instances of CFSE protruding into another cell were analyzed independently by 3 individuals using ImageJ software. Any instances in which individuals disagreed on whether transfer was occurring were rejected. Data are represented as the mean of each researcher’s analysis. Data were quantified using the following formula:


$$\frac{Synapse\;Integrin\;MFI}{Synapse\;WGA\;MFI}/\frac{Whole\;Cell\;Integrin\;MFI}{Whole\;Cell\;WGA\;MFI}$$

The ratio of WGA to antibody was used to account for differences in membrane quantity while this ratio at the synapse was compared to that of the whole cell to assess potential enrichment at the transfer site. A result of 1 was interpreted as comparable staining intensity between transfer site and cell. A result above 1 was interpreted as enriched integrin at the site of transfer.

To ensure that our results were not due to fluorescence bleed-through of the CFSE, 2 replicates were conducted using WGA conjugated to AF647 and TRITC-conjugated secondary antibody to label the appropriate integrin while the third replicate utilized TRITC-conjugated WGA and AF647-conjugated secondary antibody. The only exception to this was use of AF647-conjugated anti-CD44, which was bright enough that only a primary antibody was used.

### Mass spectrometry analysis

Surface proteins from *F. tularensis*-infected or uninfected J774A.1 cells were collected using Pierce Cell Surface Protein Isolation Kit (Thermo Scientific, Cat 89881) according to the manufacturer’s protocol. Recovered peptides were analyzed by Orbitrap LC-MS and MaxQuant software (Max Planck Institute of Biochemistry). Hits differentially expressed in infected cells with known cell-to-cell interaction activities were selected for further experimentation.

### Mouse institutional approval

All mice were handled according to Office of the Campus Veterinarian Protocol (#04946) and approved by the Institutional Care and Use Committee at Washington State University.

### Statistics

Statistical analyses indicated in individual figure legends. Statistics were determined using Graphpad Prism software. All samples for assays were included.

## Data Availability

The original contributions presented in the study are included in the article/**Supplementary Material**. Further inquiries can be directed to the corresponding author.

## Supplementary material

Supplementary Figure 1 Bacteria-stimulated merocytophagy requires cell-cell contact. **(A)** Diagram of assay. **(B)** The difference in Calcein transfer in the recipient CTR cells normalized to the uninfected sample for each group. Data from 3 independent experiments performed in triplicate. Mean +/- SD. Unpaired t test. Ns – not significant, *** p<0.001.

Supplementary Figure 2 Pathogens increase the average amount of Calcein acquired by each recipient cell. Y-axis represents the geometric mean fluorescence intensity of Calcein in recipient cells normalized to uninfected control. **(A–D)** Calcein-labeled BMDMs were infected with the indicated strain and co-incubated with recipient cells for 6 hours. Results normalized due to fluctuations in Calcein labeling and transfer. Data from 3 independent experiments performed in triplicate. Mean +/- SD. Unpaired t-test. *** p<0.0001.

Supplementary Figure 3 Salmonella debris stimulates merocytophagy. Calcein-labeled BMDMs were co-incubated with CTR-stained recipient cells with the supernatant from boiled *S.* Typhimurium for 6 hours. Y-axis represents the geometric mean fluorescence intensity of Calcein in recipient cells normalized to uninfected control. Data from 3 independent experiments performed in triplicate. Mean +/- SD. Unpaired t-test. *** p<0.0001.

Supplementary Figure 4 Titration of TLR agonists. **(A)** The percent of recipient BMDMs that acquired Calcein during 6 hour co-incubation with the indicated concentration of agonist. One-way ANOVA with a Dunnett post-test. Ns- not significant. *** p<0.0001. **(B)** Calcein transfer kinetics after exposure to LPS. Unpaired t test. *** p<0.0001. Mean +/- SD. Data from 3 independent experiments performed in triplicate.

Supplementary Figure 5 PI3K activity contributes to cytosolic transfer by merocytophagy. The percent of recipient J774A.1 macrophages (CFSE^+^) which acquired non-specific protein from a CTR-labeled donor population after 6hrs of cell-to-cell contact. Both donor and recipient populations were treated with 10μM Wortmannin or 0.1% DMSO as vehicle control for the duration of contact with no pre-treatment. Data represent mean +/- SD from 3 independent experiments conducted in technical triplicate. Unpaired t-test. Ns- not significant. ** p<0.001.

Supplementary Figure 6 Infection frequency of donor cells 24 hours post-inoculation. **(A, B)** The percent of donor cells infected after a 24 hour infection and 6 hour co-incubation with recipient cells. From 3 independent experiments performed in triplicate. Mean +/- SD. Unpaired t-test. Ns- not significant. *** p<0.0001.

Supplementary Figure 7 Verification of integrin knockdown. **(A, B)** The mean fluorescence intensity of surface expression of the indicated integrin. X-axis indicates time post-transfection. Represented as mean +/- SD.

Supplementary Figure 8 Cell-to-cell contact enhances viability of infected BMDMs. BMDM were seeded at 100,000 per well in a 24-well plate and infected with *F. tularensis* Schu S4 (MOI 100) for 18 hours. 125,000 CTR-stained BMDM were added on top of infected population for 6 hours. Cell viability of CTR-negative populations were assessed by staining (Pacific Blue Succinimidyl Ester) and flow cytometry and compared to population with no cells added. Represented as mean +/- SD, Unpaired t-test. Ns- not significant. * p<0.01. ** p<0.001.
