# Supplementary material for: Merocytophagy is an integrin-stabilized macrophage response to microbes reliant on Syk signaling
Source: Front Immunol. 2025 Apr 17;16:1565250. doi: 10.3389/fimmu.2025.1565250 (PMC12043706; doi:10.3389/fimmu.2025.1565250)
Supplement: Supplementary Figure 1 — Bacteria-stimulated merocytophagy requires cell-cell contact. (A) Diagram of assay. (B) The difference in Calcein transfer in the recipient CTR cells normalized to the uninfected sample for each group. Data from 3 independent experiments performed in triplicate. Mean +/- SD. Unpaired t test. Ns – not significant, *** p<0.001. [file DataSheet1.docx]

***Supplemental Figures***


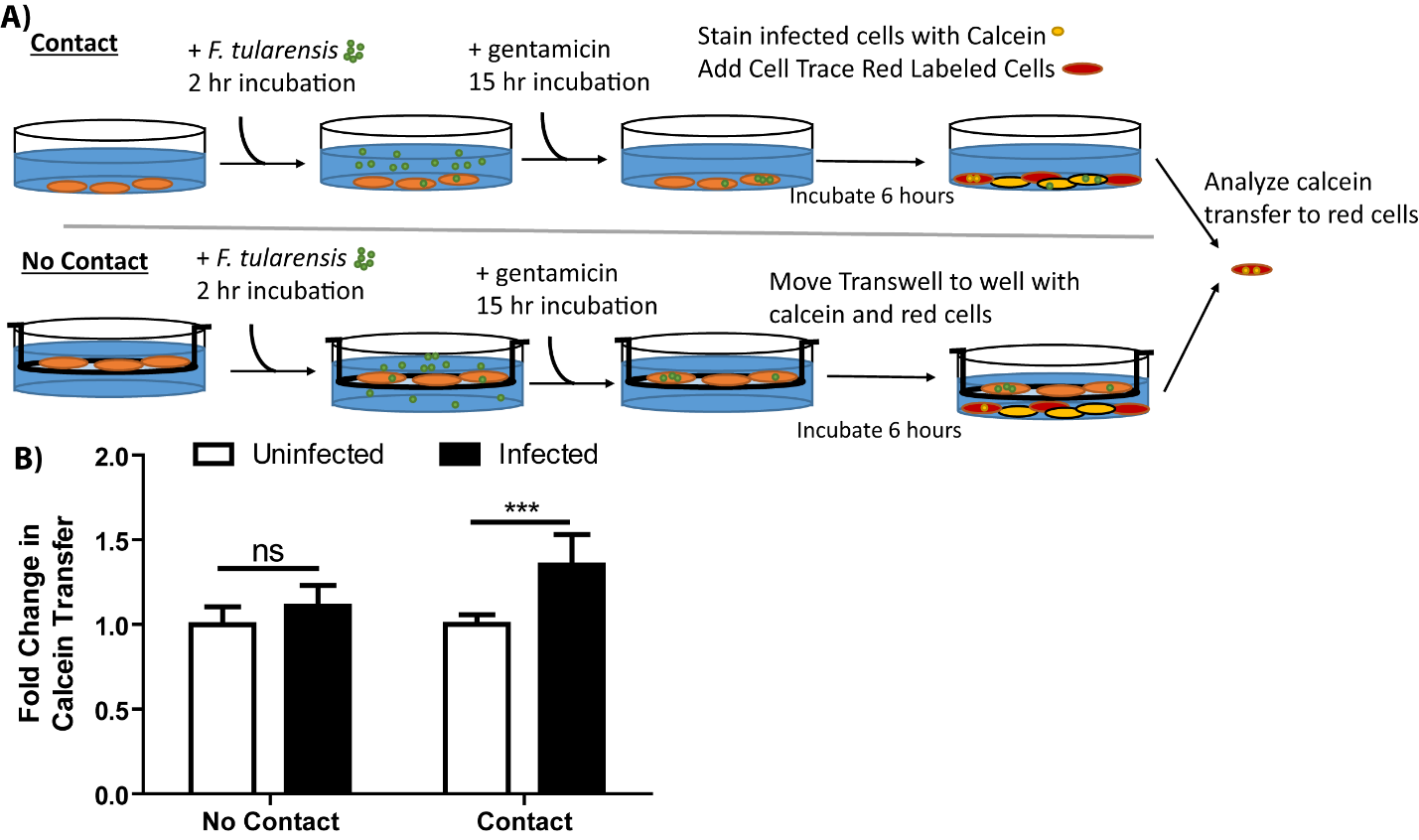


***Supplemental Figure 1:*** ***Bacteria-stimulated merocytophagy requires cell-cell contact.* A)** Diagram of assay. **B)** The difference in Calcein transfer in the recipient CTR cells normalized to the uninfected sample for each group. Data from 3 independent experiments performed in triplicate. Mean +/- SD. Unpaired t test. Ns – not significant, *** p<0.001.


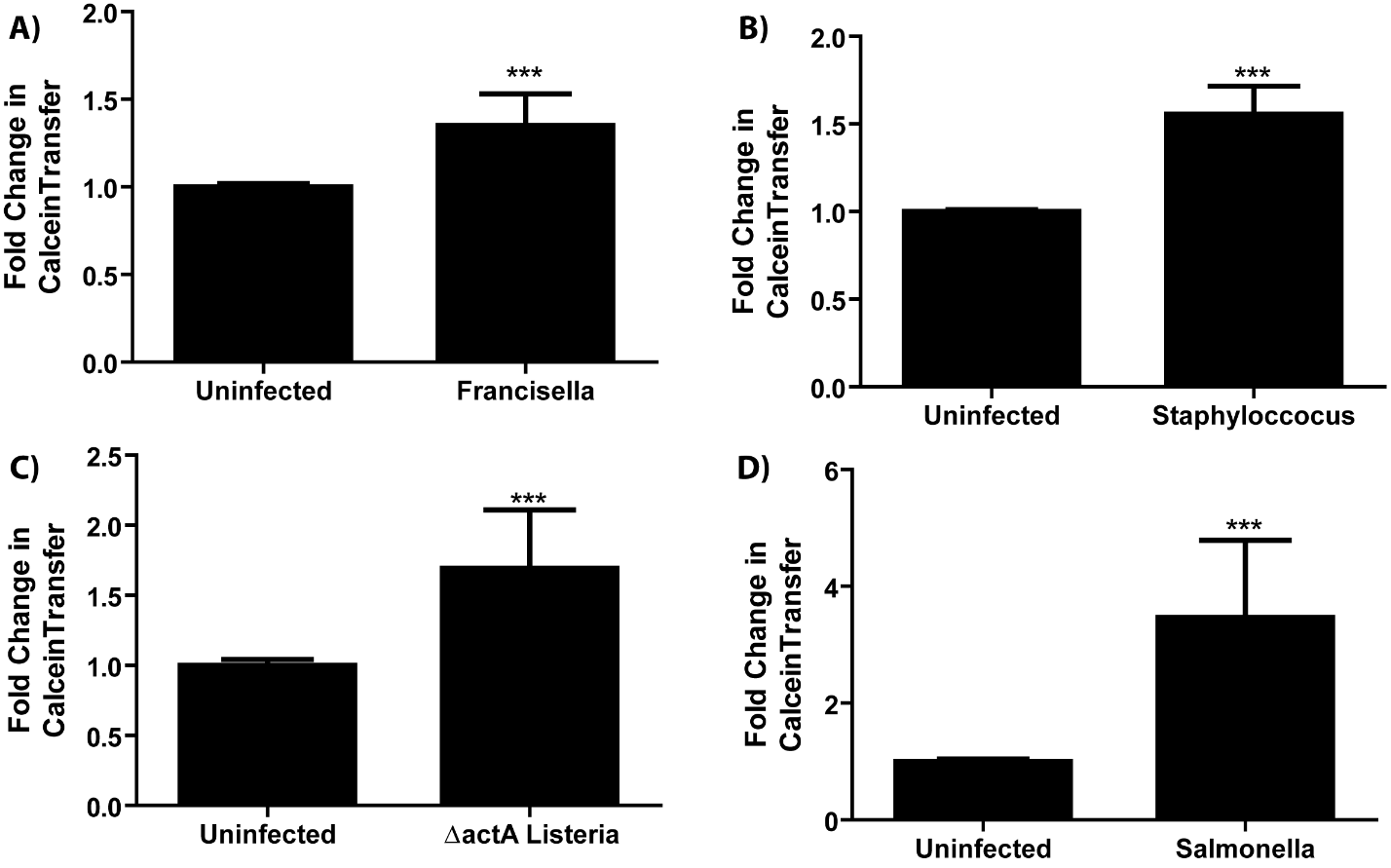


***Supplemental Figure 2: Pathogens increase the average amount of Calcein acquired by each recipient cell.*** Y-axis represents the geometric mean fluorescence intensity of Calcein in recipient cells normalized to uninfected control. ***A-*D)** Calcein-labeled BMDMs were infected with the indicated strain and co-incubated with recipient cells for 6 hours. Results normalized due to fluctuations in Calcein labeling and transfer. Data from 3 independent experiments performed in triplicate. Mean +/- SD. Unpaired t-test. *** p<0.0001.


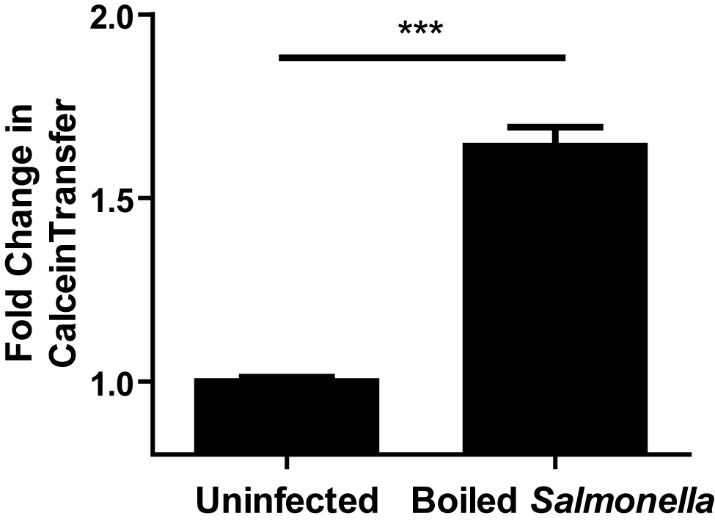


***Supplemental Figure 3: Salmonella debris stimulates merocytophagy.*** Calcein-labeled BMDMs were co-incubated with CTR-stained recipient cells with the supernatant from boiled *S.* Typhimurium for 6 hours. Y-axis represents the geometric mean fluorescence intensity of Calcein in recipient cells normalized to uninfected control. Data from 3 independent experiments performed in triplicate. Mean +/- SD. Unpaired t-test. *** p<0.0001.

**
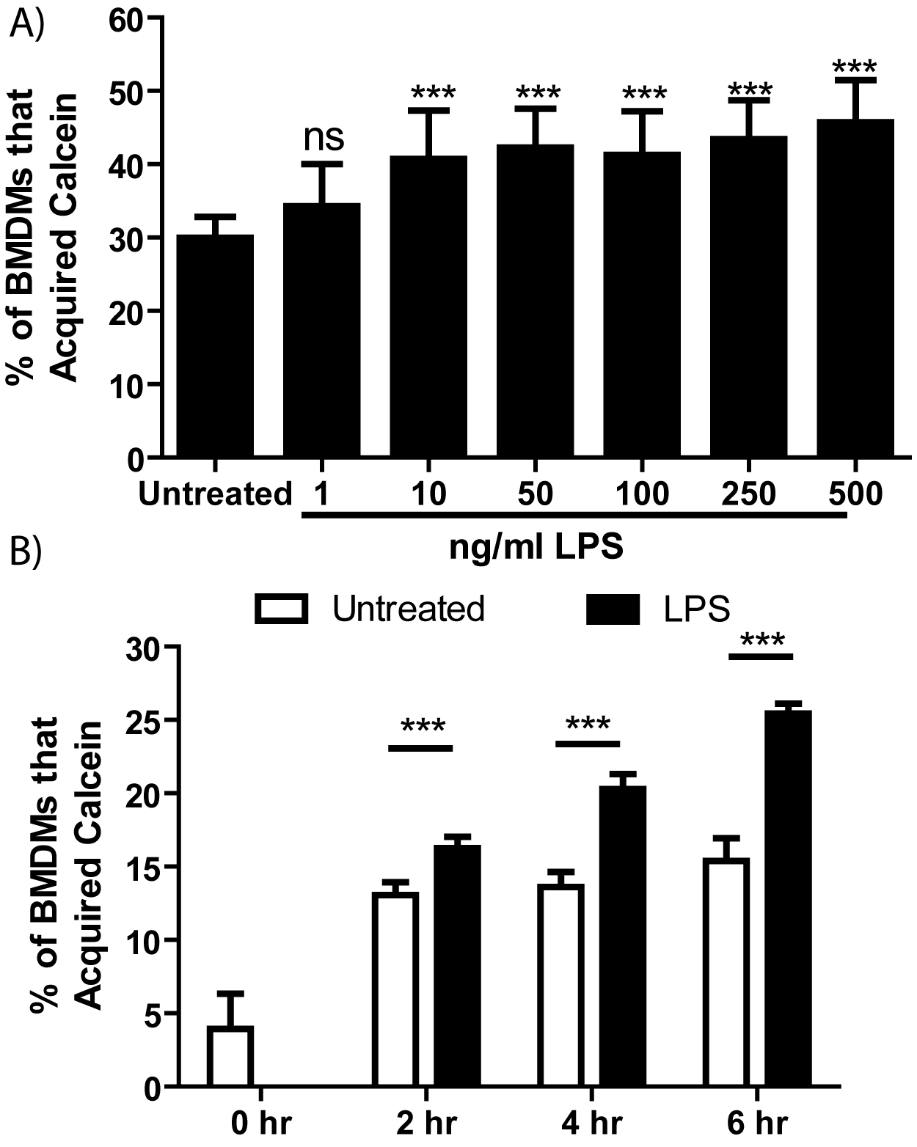
**

***Supplemental Figure 4: Titration of TLR agonists.* A)** The percent of recipient BMDMs that acquired Calcein during 6 hour co-incubation with the indicated concentration of agonist. One-way ANOVA with a Dunnett post-test. Ns- not significant. *** p<0.0001. **B)** Calcein transfer kinetics after exposure to LPS. Unpaired t test. *** p<0.0001. Mean +/- SD. Data from 3 independent experiments performed in triplicate.

***Supplemental Figure 5: PI3K activity contributes to cytosolic transfer by merocytophagy.*** The percent of recipient J774A.1 macrophages (CFSE^+^) which acquired non-specific protein from a CTR-labeled donor population after 6hrs of cell-to-cell contact. Both donor and recipient populations were treated with 10μM Wortmannin or 0.1% DMSO as vehicle control for the duration of contact with no pre-treatment. Data represent mean +/- SD from 3 independent experiments conducted in technical triplicate. Unpaired t-test. Ns- not significant. ** p<0.001.


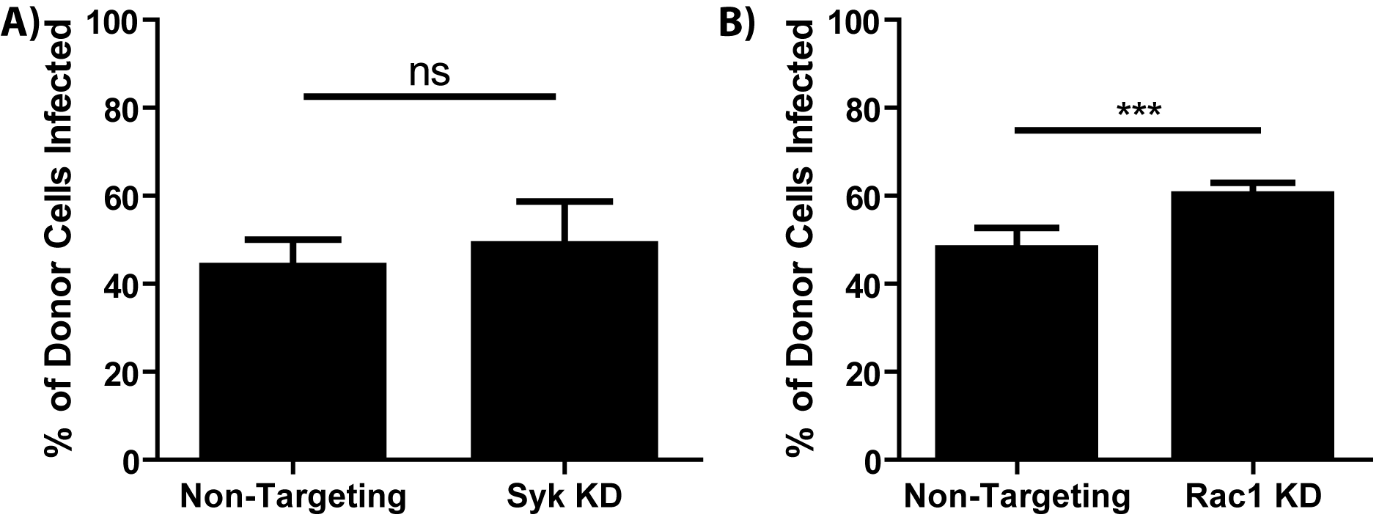


***Supplemental Figure 6: Infection frequency of donor cells 24 hours post-inoculation.* A-B)** The percent of donor cells infected after a 24 hour infection and 6 hour co-incubation with recipient cells. From 3 independent experiments performed in triplicate. Mean +/- SD. Unpaired t-test. Ns- not significant. *** p<0.0001.


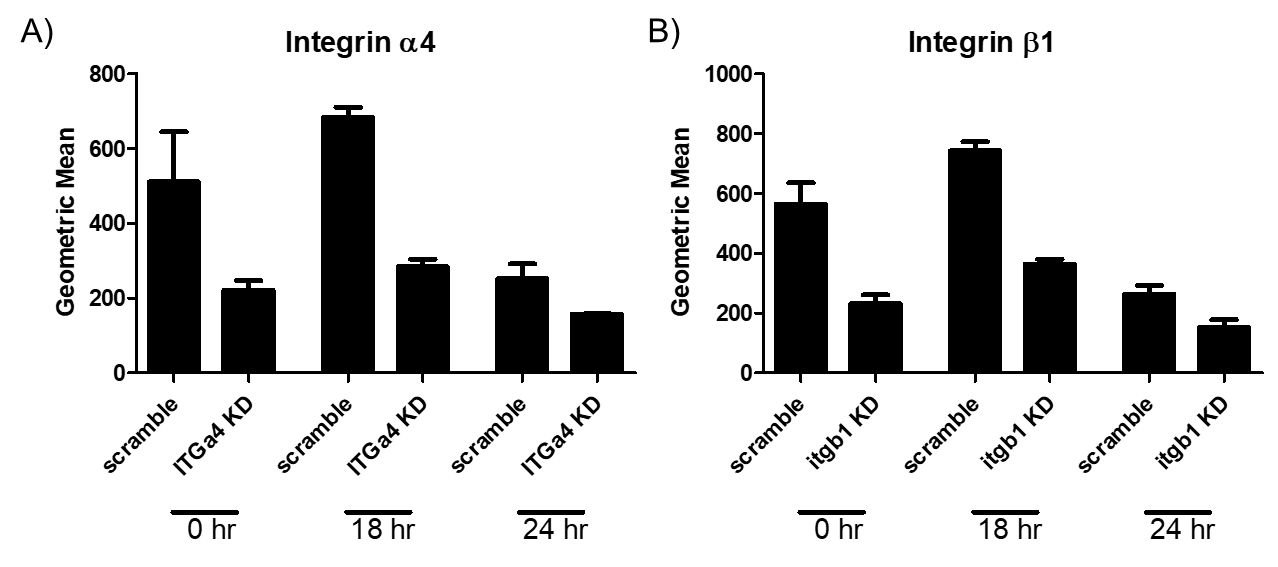


***Supplemental Figure 7: Verification of integrin knockdown.* A-B)** The mean fluorescence intensity of surface expression of the indicated integrin. X-axis indicates time post-transfection. Represented as mean +/- SD.

***Supplemental Figure 8: Cell-to-cell contact enhances viability of infected BMDMs.*** BMDM were seeded at 100,000 per well in a 24-well plate and infected with *F. tularensis* Schu S4 (MOI 100) for 18 hours. 125,000 CTR-stained BMDM were added on top of infected population for 6 hours. Cell viability of CTR-negative populations were assessed by staining (Pacific Blue Succinimidyl Ester) and flow cytometry and compared to population with no cells added. Represented as mean +/- SD, Unpaired t-test. Ns- not significant. * p<0.01. ** p<0.001.
